# Tracing halogen and B cycling in subduction zones based on obducted, subducted and forearc serpentinites of the Dominican Republic

**DOI:** 10.1038/s41598-017-18139-7

**Published:** 2017-12-19

**Authors:** Lilianne Pagé, Keiko Hattori

**Affiliations:** Department of Earth and Environmental Sciences, University of Ottawa, Ottawa, K1N 6N5 Canada

## Abstract

Serpentinites are important reservoirs of fluid-mobile elements in subduction zones, contributing to volatiles in arc magmas and their transport into the Earth’s mantle. This paper reports halogen (F, Cl, Br, I) and B abundances of serpentinites from the Dominican Republic, including obducted and subducted abyssal serpentinites and forearc mantle serpentinites. Abyssal serpentinite compositions indicate the incorporation of these elements from seawater and sediments during serpentinization on the seafloor and at slab bending. During their subduction and subsequent lizardite-antigorite transition, F and B are retained in serpentinites, whilst Cl, Br and I are expelled. Forearc mantle serpentinite compositions suggest their hydration by fluids released from subducting altered oceanic crust and abyssal serpentinites, with only minor sediment contribution. This finding is consistent with the minimal subduction of sediments in the Dominican Republic. Forearc mantle serpentinites have F/Cl and B/Cl ratios similar to arc magmas, suggesting the importance of serpentinite dehydration in the generation of arc magmatism in the mantle wedge.

## Introduction

Subduction of hydrated lithologies provides a mechanism for recycling fluid-mobile elements (FME) from surface reservoirs, such as the hydrosphere, atmosphere and sediments, into the Earth’s mantle. The high abundance of volatiles, such as halogens and B, in arc magmas^1,2^, attest to their recycling at convergent plate boundaries. Serpentinites, primarily composed of the mineral serpentine, are an important component of the volatile budget in subduction zones because they contain up to 13 wt% H_2_O and are stable to >100 km depth^3^, making them effective vehicles for the transport of FME into the mantle. Recent studies of abyssal and forearc mantle serpentinites have demonstrated their importance as reservoirs for halogens and B in subduction zones^4–12^. However, the role of serpentinites in the recycling of these elements is not fully understood. Halogen concentrations and ratios can offer a tool for tracking fluids through subduction zones. For example, the halogen ratios of forearc mantle serpentinites may provide insight into the make-up of the slab in a given subduction complex, and help elucidate the extent to which abyssal serpentinites, altered oceanic crust, and sediments contribute to slab-derived fluids. The first study of all four halogens in abyssal serpentinites and their subducted counterparts was conducted on the serpentinized Erro-Tobbio peridotites of the Ligurian Alps^13^. Our paper expands on this work by presenting the halogen and B contents of Dominican Republic (DR) serpentinites, including those of abyssal origin and of the forearc mantle, with the purpose of evaluating the behaviour and fractionation of these elements in subduction zones.

## Samples

**Figure 1:**
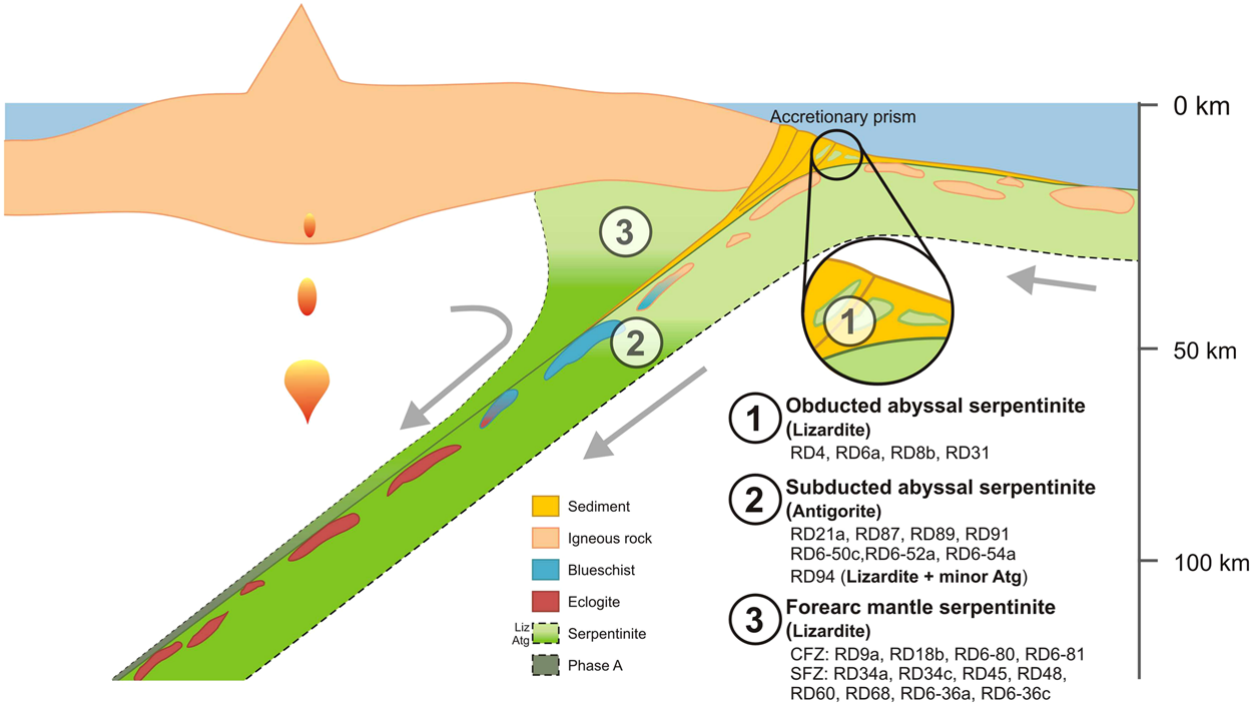
Schematic cross-section of a subduction complex showing position of serpentinite samples from the Dominican Republic: (1) obducted abyssal lizardite, (2) subducted abyssal antigorite (~50 km depth), and (3) forearc mantle lizardite (<35 km depth). The incoming plate formed at an ultra-slow spreading ridge was composed of abyssal peridotites with minor oceanic basalts and overlying sediments. The upper portion of the slab was obducted forming an accretionary prism. Fluids released from the slab hydrated the overlying peridotites to form forearc mantle serpentinites. Liz – lizardite, Atg – antigorite.

Serpentinite samples were collected from the Tertiary subduction complex in northern Dominican Republic on the island of Hispaniola. This complex was formed by the subduction of the Proto-Caribbean oceanic lithosphere beneath the NE-migrating Caribbean plate. The former was produced at an ultra-slow spreading ridge^14^, and, as such, it was primarily composed of abyssal peridotites with minor cover of basaltic rocks and sediments. During subduction, the upper part of this young oceanic lithosphere was scraped off to form an accretionary prism on the northern part of the island, while the remaining slab was subducted (Fig. 1). The subduction complex includes the Puerto Plata Complex (PPC) and the Rio San Juan Complex (RSJC)^15^.

The DR serpentinite samples were previously characterized on the basis of their occurrences and petrographical and geochemical data^16^. They are classified into three types: i) obducted abyssal ii) subducted abyssal, and iii) forearc mantle serpentinites (Fig. 1). Abyssal serpentinites are formed by the hydration of abyssal peridotites near oceanic ridges and on the seafloor^17^, or at the outer rise by fluid infiltration along extensional faults in the bending slab^18^. Obducted abyssal serpentinites are in the accretionary prism (Fig. 1), and have not been subducted. Forearc mantle serpentinites are hydrated by fluids released from the subducting slab^17^. Detailed descriptions of samples and their protoliths are given in Supplementary Information.

Samples representing obducted abyssal serpentinites were collected from the PPC and the northern RSJC (RD31, RD4, RD6a, RD8b). They are comprised of lizardite (low temperature serpentine phase) pseudomorphically replacing olivine and orthopyroxene. Samples representing subducted abyssal serpentinites were collected from the mélanges of Arroyo Sabana (RD87, RD89) and Jagua Clara (RD21a, RD91, RD94, RD650c, RD6-52a, RD6-54a) of the RSJC. They are composed of antigorite (high temperature phase), with minor chlorite, talc and tremolite, except for RD94 that is predominantly lizardite with minor antigorite blades. Lizardite changes to antigorite between 320 and 390 °C^19^. Using the geothermal gradient of this subduction zone (8 °C/km)^20^, the depth of lizardite-bearing sample RD94 is estimated to be ~40–50 km, whereas, the remaining antigorite-only serpentinites come from depths >50 km. A lack of metamorphic olivine in these samples further constrains their depth to <80 km, given its crystallization from antigorite at 650 °C^21^.

Samples representing shallow forearc mantle lizardite-serpentinites were collected from boulders along the stream of Rio Cuevas (RD45, RD48, RD60) and from the serpentinite lenses along two major strike-slip fault zones: the Septentrional fault zone (RD34a, RD34c, RD68, RD6-36a, RD6-36c) and the Camú fault zones (RD9a, RD18b, RD6-80, RD6-81). The lack of antigorite in these samples constrains their depth to <40 km. A forearc mantle origin of these samples is indicated by low Al and high Ir-type platinum group element concentrations in the bulk rocks and high Cr in relict spinel^16^.

## Results

### Halogens

**Figure 2:**
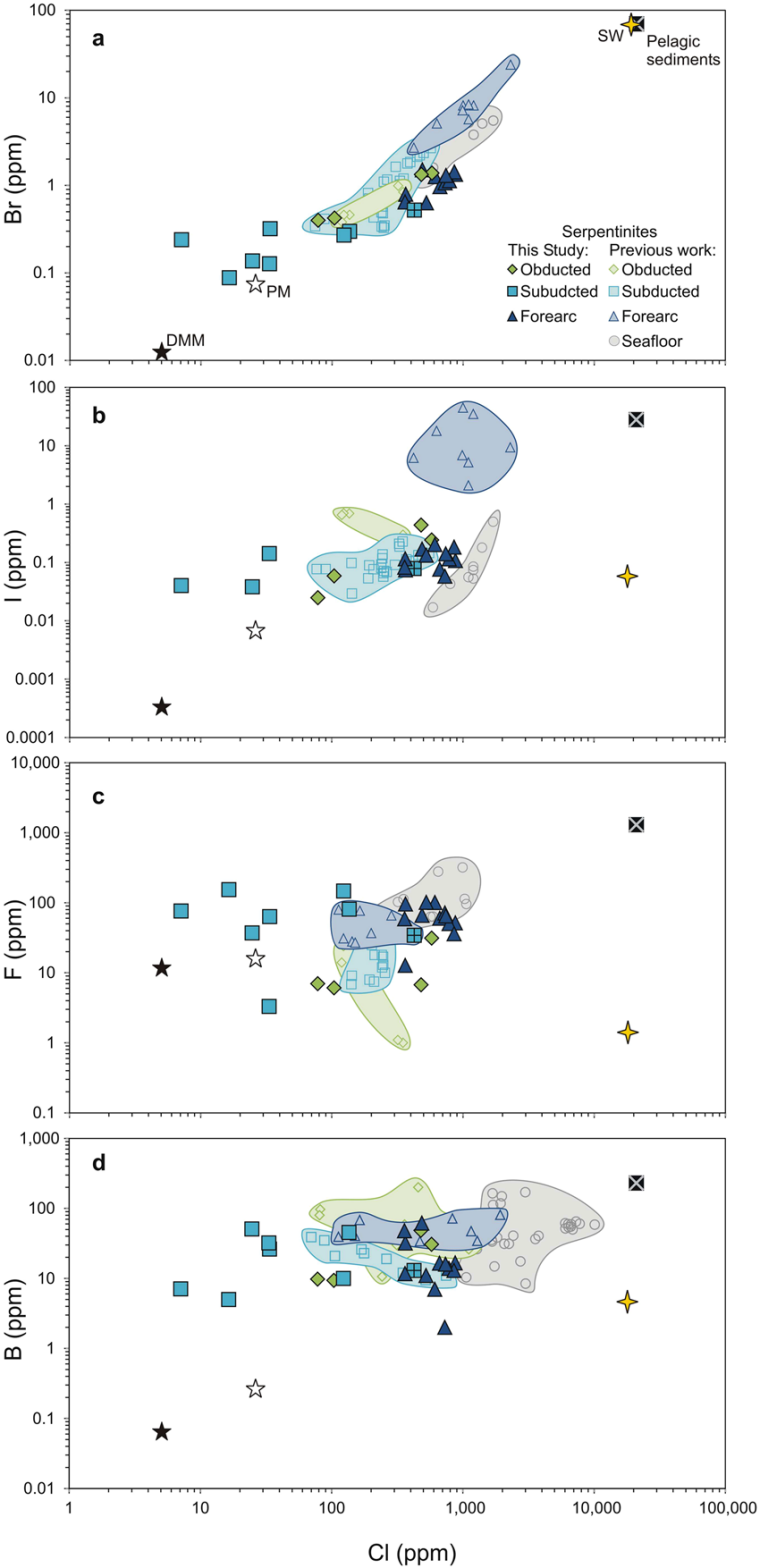
Halogen and B concentrations of abyssal and forearc serpentinites from the Dominican Republic. (**a**) Bromine and Chlorine show a broad positive correlation along a linear trend with the depleted MORB mantle (DMM)^22^, primitive mantle (PM)^22^ and seawater (SW)^25^. Data for the DR obducted abyssal lizardite-serpentinites overlap with those of other pre-subduction serpentinites elsewhere^13^, and Cl contents of the DR forearc lizardite-serpentinites are similar to those of other forearcs, such as Mariana and Guatemala^8^. The subducted serpentinites show wide ranges in Cl and Br contents, with the antigorite-bearing samples displaying lower concentrations than lizardite-bearing sample RD94. (**b**) The I and Cl values of all samples lie along the linear trend defined by DMM, PM and average pelagic sediment^30^ values, except for the antigorite-samples. (**c**) Fluorine and Chlorine do not appear to correlate across the three sample sets. Data for all DR serpentinites, except the antigorite-bearing samples, plot within the triangle defined by seawater, the DMM, and pelagic sediment. (**d**) Boron concentrations are similar among all samples and literature data.

**Figure 3:**
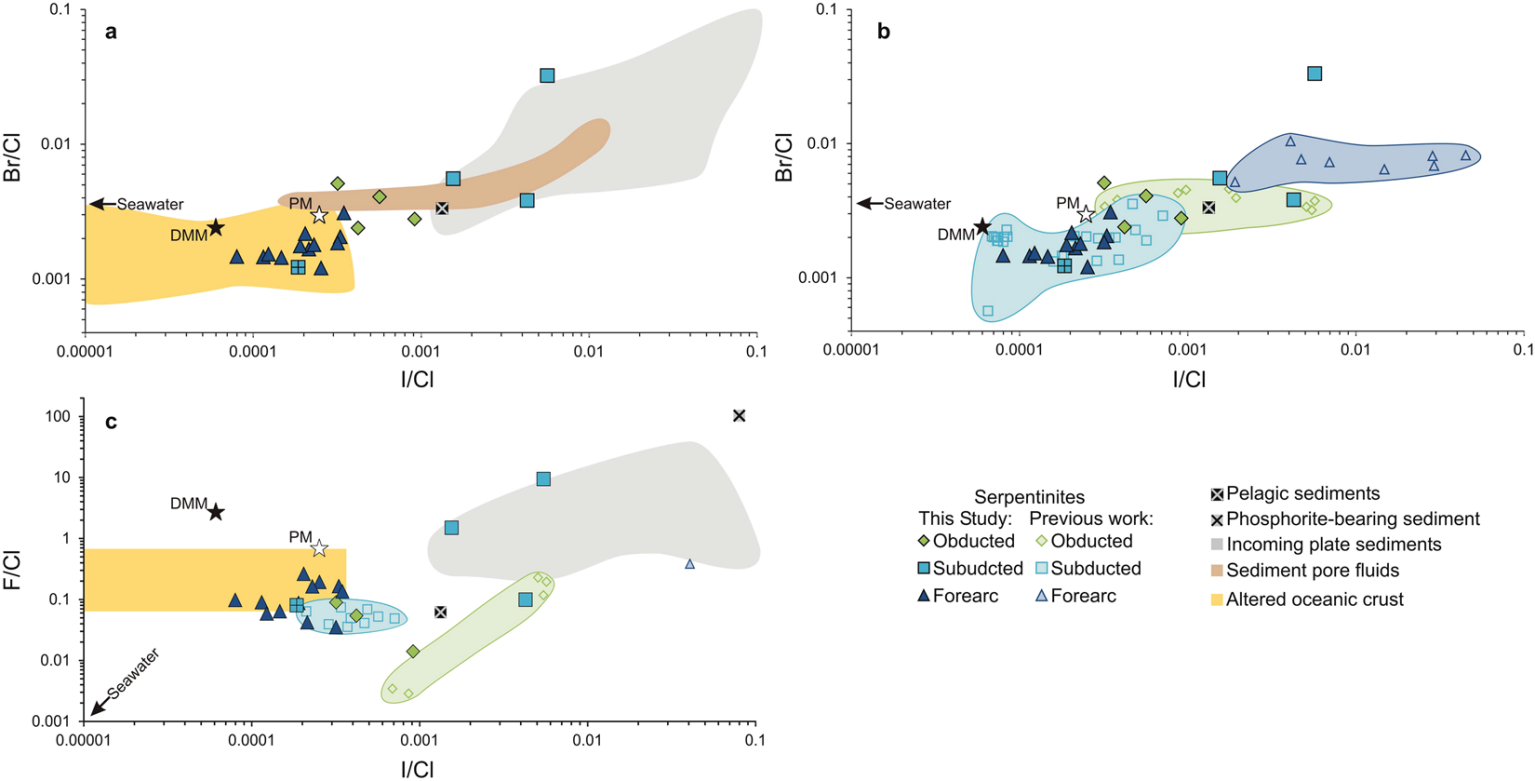
Halogen ratios of abyssal and forearc serpentinites from the Dominican Republic are compared to other rock types and serpentinite data from elsewhere. (**a**) There is a broad correlation between Br/Cl and I/Cl ratios among all samples and lithologies. The DR obducted abyssal lizardite-serpentinites have Br/Cl and I/Cl ratios that overlap with the lower end of sedimentary marine pore fluids^55,56^, while their subducted antigorite-bearing counterparts have ratios that extend to higher values (except for lizardite-bearing RD94). The forearc lizardite-serpentinites have the lowest Br/Cl and I/Cl ratios and overlap with values for altered oceanic crust^39^. (**b**) The DR obducted lizardite-serpentinites are similar to other pre-subduction serpentinites, whereas the DR forearc samples have lower ratios than other forearc serpentinites, such as those from Mariana and Guatemala. (**c**) The F/Cl ratios of the DR obducted abyssal and forearc mantle serpentinites are similar to those of other lizardite-bearing serpentinites, and the forearc samples overlap with altered oceanic crust values^57^. The DR subducted antigorite-serpentinites have higher F/Cl ratios (Table 1) than other antigorite-bearing samples and overlap with incoming sediments. References for SW, DMM, PM and plate sediments are the same as in Fig. 2.

For each sample, bulk halogen data are obtained using pyrohydrolysis extraction with ion chromatography (F, Cl) and ICP-MS (Br, I), and bulk B data are obtained by prompt gamma neutron activation analysis (Table 1; Fig. 2). The obducted abyssal lizardite-serpentinites display two different halogen signatures (Table 1; Fig. 2). Samples RD4 and RD6a contain higher concentrations of Cl (480–580 ppm), Br (1.3–1.4 ppm) and I (0.24–0.44 ppm) compared to RD31 and RD8b (78–100 ppm Cl, 0.40–0.42 ppm Br, 0.025–0.059 ppm I). Fluorine contents are relatively low in all obducted abyssal serpentinites (6.1–31 ppm). Ratios of F/Cl, Br/Cl and I/Cl for these samples range from 0.014 to 0.089, 0.0024 to 0.0051 and 0.00032 to 0.00091, respectively (Fig. 3).

**Table 1:** Bulk halogen and B contents (ppm) of serpentinites from the northern Dominican Republic, as determined by ICP-MS (Br, I) and IC (F, Cl) following pyrohydrolysis extractions and PGNAA (B).

Sample	Serpentine phase	F	Cl	Br	I	B	F/Cl	Br/Cl	I/Cl	B/Cl
n = 2 or 3		ppm	ppm	ppm	ppm	ppm				
Obducted abyssal										
RD4	lizardite	31	578	1.4	0.24	30.7	0.054	0.0024	0.0004	0.05
RD6a	lizardite	6.8	480	1.3	0.44	48.5	0.014	0.0028	0.0009	0.10
RD8b	lizardite	7.0	78	0.40	0.025	9.8	0.089	0.0051	0.0003	0.13
RD31	lizardite	6.1	104	0.42	0.059	9.4	0.059	0.0041	0.0006	0.09
*Average*		*13*	*310*	*0.89*	*0.19*	*24.6*	*0.05*	*0.0036*	*0.0006*	*0.09*
Subducted abyssal										
*RD6–50c*	antigorite	76	7	0.24	0.040	7.1	10.7	0.034	0.0057	1.00
RD6–52a	antigorite	64	34	0.32	bdl	26.3	1.9	0.010	bdl	0.78
RD6–54a	antigorite	37	25	0.14	0.038	51.0	1.5	0.0055	0.0016	2.06
RD21a	antigorite	3	33	0.13	0.142	32.2	0.1	0.0038	0.0043	0.97
RD87	antigorite	81	136	0.30	bdl	45.4	0.6	0.0022	bdl	0.33
RD89	antigorite	147	123	0.27	bdl	10.0	1.2	0.0022	bdl	0.08
RD91	antigorite	154	16	0.088	bdl	5.0	9	0.005	bdl	0.30
RD94	lizardite	34	425	0.52	0.0788	13.0	0	0.001	0.0002	0.03
Average*		*80*	*54*	*0.21*	*0.074*	*25.3*	*3.63*	*0.0089*	*0.0038*	*0.79*
Forearc mantle										
Septentrional Fault Zone										
RD34a	lizardite	101	612	1.3	0.20	7.0	0.17	0.0021	0.0003	0.01
RD34c	lizardite	60	666	0.97	0.08	16.5	0.090	0.0015	0.0001	0.02
RD45	lizardite	72	729	1.1	0.058	2.0	0.098	0.0015	0.0001	0.00
RD48	lizardite	51	785	1.1	0.116	13.9	0.064	0.0014	0.0001	0.02
RD60	lizardite	52	876	1.3	0.11	16.7	0.059	0.0015	0.0001	0.02
RD68	lizardite	102	525	0.6	0.13	11.0	0.194	0.0012	0.0003	0.02
RD6–36a	lizardite	36	856	1.4	0.18	13.0	0.042	0.0017	0.0002	0.02
RD6–36c	lizardite	65	739	1.3	0.14	16.0	0.088	0.0018	0.0002	0.02
Camú Fault Zone										
RD9A	lizardite	96	365	0.79	0.07	32.0	0.26	0.0022	0.0002	0.09
RD18B	lizardite	66	488	1.5	0.17	61.6	0.14	0.0031	0.0003	0.13
RD6–80	lizardite	59	360	0.6	0.08	48.0	0.16	0.0018	0.0002	0.13
RD6–81	lizardite	13	363	0.671	0.12	11.7	0.035	0.0018	0.0003	0.03
*Average*		*64*	*614*	*1.1*	*0.12*	*21*	*0.15*	*0.0022*	*0.0003*	*0.09*

*Average excludes lizardite-bearing RD94.

bdl - below detection limit.

The subducted antigorite-serpentinites have much lower Cl (7.1–140 ppm) and Br (0.088–0.32 ppm) concentrations than their obducted lizardite-bearing counterparts. The one exception is RD94, a subducted lizardite-bearing sample that contains similarly high concentrations of Cl (430 ppm) and Br (0.52 ppm) (Fig. 2). Iodine concentrations are only reported for four of the eight subducted serpentinites due to the higher I blank of the pyrohydrolysis extraction method (see Methods for details). These concentrations (0.038–0.14 ppm) are similar to those of the obducted samples. Fluorine contents of most of the subducted serpentinites are elevated (up to 150 ppm) relative to the obducted samples. The Br/Cl and I/Cl ratios of lizardite-bearing RD94 (0.0012 and 0.00019, respectively) are lower than those of the obducted lizardite-serpentinites, whereas the Br/Cl and I/Cl ratios of the remaining subducted antigorite-serpentinites are higher and cover a larger range (0.0016–0.0057 and 0.0022–0.034, respectively; Fig. 3b). The F/Cl ratio of RD94 (0.080) is also similar to those of the obducted serpentinites, while the ratios of other subducted serpentinites are up to two orders of magnitude greater, and they cover a wide range (0.10–11; Fig. 3c).

The lizardite-serpentinites from the shallow forearc mantle have high heavy halogen concentrations (360–880 ppm Cl, 0.63–1.5 ppm Br, 0.058–0.20 ppm I), similar to those of the obducted abyssal lizardite-serpentinites (Fig. 2a,b), but their Br/Cl and I/Cl ratios (0.0012–0.0031 and 0.00008–0.00035, respectively) trend towards lower values (Fig. 3b). Their Br/Cl and I/Cl ratios are also much lower than those of the antigorite-serpentinites of the slab, but are similar to those of the subducted lizardite-bearing sample. Fluorine concentrations are also high in the forearc mantle lizardite-serpentinites (13–100 ppm) and similar to those of the subducted antigorite-serpentinites (Fig. 2c). Yet, their F/Cl ratios (0.035–0.26) are much lower due to elevated Cl contents in the serpentinized mantle. The F/Cl ratios of the forearc mantle serpentinites are similar to those of the obducted abyssal lizardite-serpentinites (Fig. 3c).

### Boron

**Figure 4:**
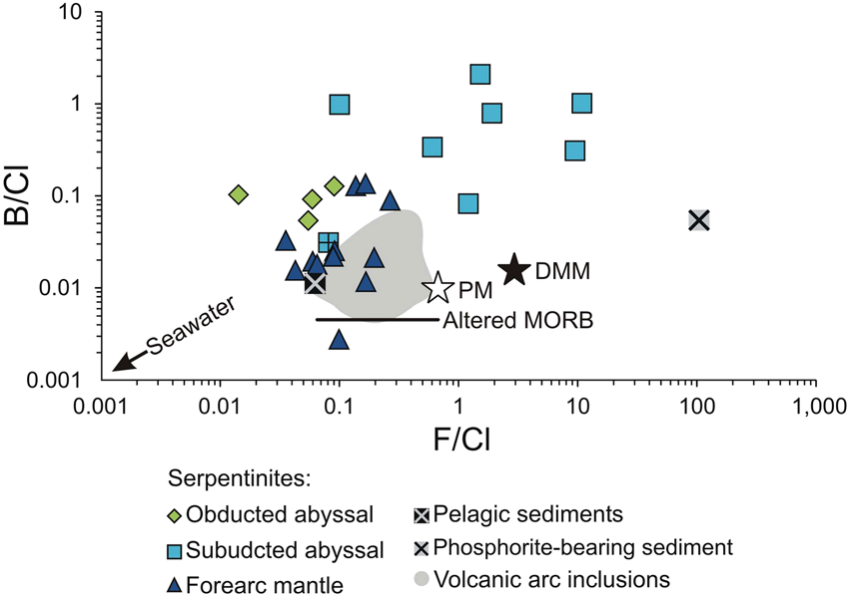
B/Cl and F/Cl ratios of serpentinites from the Dominican Republic are shown along with altered oceanic crust^57,58^, average pelagic sediment^30^ and volcanic melt inclusions^1,2^. The B/Cl and F/Cl ratios of the forearc lizardite-serpentinites overlap with those of the melt inclusions, while those of the subducted antigorite-serpentinites extend to much higher values. References for DMM and PM are the same as Fig. 2.

The B/Cl ratios of the obducted abyssal lizardite-serpentinites cover a narrow range (0.053–0.13; Fig. 4). Chlorine-rich samples (RD4 and RD6A) contain high B (31–49 ppm) and Cl-poor samples (RD31 and RD8b) have low B (9–10 ppm) (Table 1). Boron concentrations of the subducted (5–51 ppm B) and shallow forearc mantle (2–62 ppm B) serpentinites cover a large, yet comparable range. Their B/Cl ratios (0.03–1.0 and 0.0027–0.13, respectively) also cover large ranges, but they differ by up to an order of magnitude, reflecting the differing Cl concentrations of the two sample sets.

## Discussion

### Halogen and B uptake by abyssal serpentinites

The protoliths of the DR abyssal serpentinites are formed at an ultra slow spreading ridge^14^ with compositions between those of the primitive mantle (PM) and the depleted MORB mantle (DMM). The Cl, Br and I contents of the obducted abyssal lizardite-serpentinites are up to one order of magnitude greater than those of the PM (26 ± 8 ppm Cl; 76 ± 25 ppb Br; 7 ± 4 ppb I)^22^ and two orders of magnitude greater than those of the DMM (5 ± 2 ppm Cl, 13 ± 6 ppb Br, 0.3 ± 0.1 ppb I)^22^ (Fig. 2a,b). Their B concentrations are also elevated, exceeding those of the PM (0.30 ppm)^23^ by two orders of magnitude and the DMM (0.06 ppm)^24^ by up to three (Fig. 2d). Seawater provides Cl and Br to account for the observed enrichment, as supported by Br/Cl ratios (avg. 0.0029) similar to that of seawater (0.0037)^25^ (Fig. 3a). In contrast, I/Cl ratios are up to 270 times greater than that of seawater, overlapping with pore fluids in marine sediments (Fig. 3a), and B/Cl ratios are up to 510 times greater than seawater. Given the high I (1.6–20 ppm)^26^ and B (96–490 ppm)^27^ contents of pelagic sediments, the I- and B-enrichment of abyssal serpentinites is attributed to a contribution of sediment-modified seawater for serpentinization. Similar halogen and B contents have been obtained for other obducted abyssal serpentinites, such as those of the Northern Apennines and the Ligurian Alps, Italy^5,8,13^ (Fig. 2).

Chlorine and Br concentrations of the obducted abyssal lizardite-serpentinites from the Dominican Republic and elsewhere (e.g., Ligurian Alps) are lower than present-day seafloor serpentinites, such as those from mid-oceanic ridges and passive continental margins (460–1700 ppm Cl and 1.3–6.8 ppm Br)^8^ (Fig. 2a), but their Br/Cl ratios are very similar (Fig. 3b). This may suggest a minor loss of Cl and Br during accretion. On the other hand, I and B concentrations of the obducted lizardite-serpentinites are very similar to those of the present-day abyssal serpentinites^8,10^ (Fig. 2b,d).

Bulk F contents in the DR obducted serpentinites are similar to those of the PM (17 ± 6 ppm) and the DMM (12 ± 2 ppm)^22^ (Fig. 2c). These low concentrations and associated low F/Cl ratios (<0.1) are similar to other pre-subduction lizardite-serpentinites, such as those from the Erro-Tobbio peridotite, Ligurian Alps^13^ (Fig. 3c). Abyssal peridotites at slow and ultra-slow spreading ridges are primarily hydrated by seawater, which contains very low F (<2 ppm)^25^. Therefore, such abyssal serpentinites are expected to be low in F. On the contrary, seafloor serpentinites formed near volcanic centres, such as the Logatchev hydrothermal field^28^ and the MARK area^12^ (Fig. 2c), have elevated F contents due to a contribution of magmatic fluids.

Fluorine contents of the DR subducted antigorite-serpentinites are variable (3–150 ppm F; Table 1), but overall values are greater than the DMM and elevated compared to their obducted lizardite-bearing counterparts (Fig. 2c). They also have elevated F/Cl ratios, which overlap with incoming plate sediments^13^ (Fig. 3c). The variably high F contents of the subducted serpentinites are attributed to sediments. Fluorine contents can vary widely among sediments, but they are particularly high in shallow water marine sediments, such as carbonates (up to 1700 ppm F)^29^ and phosphorites (up to 3.1 wt% F)^30^. Subduction zone trenches are filled with such shallow water sediments along with terrigenous material from volcanic arcs and pelagic sediments on the incoming plate. The pore fluids of trench sediments are likely F-rich since some minerals, such as carbonates, dissolve in deep water. We suggest the F-enrichment in subducting serpentinites is attributed to the interaction of abyssal serpentinites with these fluids.

### Halogen and B behaviour during shallow subduction

Except for the lizardite-bearing sample RD94, the Cl and Br concentrations of the subducted serpentinites are up to one order of magnitude lower than those of the obducted abyssal serpentinites, and they are among the lowest concentrations reported for subducted serpentinites in previous studies^7,13^ (Fig. 2a). These results suggest a loss of these elements during subduction and are consistent with previous reports of up to a 90% loss of Cl during the shallow (<30 km) subduction of abyssal serpentinites^5,9^. Iodine contents of the DR subducted serpentinites are similar to those of the obducted samples and to other subducted samples in the literature (Fig. 2b). Yet, considering four of the eight samples contain I concentrations below method detection limit (Table 1), the average I content of the subducted serpentinites is most likely below those of the DR obducted samples and subducted serpentinites of other studies. In all studies of paleo-subduction zone serpentinites, including this one, there is some uncertainty regarding the composition of abyssal serpentinites prior to their subduction. In this study, although we consider the obducted abyssal serpentinites to represent the composition of abyssal serpentinites prior to their subduction, the former may have been modified during obduction and therefore, may not be a true representation of the composition of incoming abyssal serpentinites. Also, variability may exist in the timing and sources of hydration. Some serpentinites may have been hydrated at the ridge or on the seafloor, whereas, some may have been hydrated by fluids dominated by sedimentary pore fluids along bend faults of the outer rise or within the trench. Varying hydration processes before subduction may explain a wide range of compositions in serpentinites from different subduction zones.

In contrast to the heavy halogens, F contents are high in subducted abyssal serpentinites. This is attributed, in part, to an input of F from F-rich sediments in the trench. The fractionation of F from the heavier halogens during subduction is also likely related to the serpentine phase change from lizardite to antigorite. This phase change at 300–350 °C is considered to be accompanied by the release of water^31^ and is conducive to the liberation of FME. In lizardite, the Si-O tetrahedra are distorted to match the octahedral sheet resulting in a flat layer structure. In antigorite, Si-O tetrahedral sheets are connected to the concave side of the continuous octahedral sheet but display periodic reversals in orientation resulting in a more wavy structure^31^. Consequently, layers in antigorite are bound through stronger, primarily covalent Si-O bonds. Antigorite’s tighter structure may not as readily accommodate larger halogens in its hydroxyl site, thus resulting in the expulsion of these larger anions during the phase transition^6^. The smaller F ion (1.33 Å), on the other hand, has a similar ionic radius to OH^−^ (1.35 Å) and is more likely to remain in the serpentine structure during the phase transition. Furthermore, F may be incorporated into the O site of serpentine by the coupled substitution of Al^3+^ and F^−^ with Si^4+^ and O^2−^ (1.21 Å), as previously suggested for F in pyroxenes^32^. This mechanism is supported by the high Al content of the antigorite-serpentinites of our study^16^.

The subducted antigorite-serpentinites of this study contain similar B contents to the obducted lizardite-bearing samples (Fig. 2d), but they have higher B/Cl ratios (Fig. 4). This indicates B is likely retained during subduction and the serpentine phase change. These results are in contrast with B loss during this transition reported by some studies^5,9^, but they are consistent with the retention of B with increasing metamorphic grade reported by others^11^. Since B is incorporated into sheet silicates by replacing tetrahedral Si or Al, it is likely retained in the mineral structure during the lizardite-antigorite phase transition. The high B concentration in the DR antigorite-serpentinites may also be the result of additional input by FME-rich fluids from trench sediments, as previously suggested to account for enrichment of other FME, such as As and Sb, in these samples^33^ (Fig. 1).

### Halogen and B uptake by forearc mantle serpentinites

Aqueous fluids are released from the slab during shallow subduction (<50 km) through compaction and dehydration of sediments and hydrous phases of the altered oceanic crust^34^. Additionally, fluids and FME may be released from underlying abyssal serpentinites during the transition from lizardite to antigorite^32,35^. A fraction of these slab-derived fluids are refluxed back to the surface along the subduction interface^36^ and some are transferred to the overlying forearc mantle to form lizardite-serpentinites at the base of the wedge^37^. Eventually, the forearc mantle serpentinites are dragged downwards by mantle corner flow and dehydrate when temperatures reach ~600–700 °C^3^. In the mantle wedge, this corresponds to depths ranging from 80 to 130 km^37^ and leads to the release of abundant fluids and FME for partial melting in the mantle wedge (Fig. 1)^37,38^. This model is supported by F/Cl and B/Cl ratios of the DR forearc mantle lizardite-serpentinites that overlap with those of magmas of the Izu^1^ and Lesser Antilles^2^ arcs (Figs 3a,c and 4).

Forearc mantle serpentinite compositions reflect those of fluids derived from sediments, oceanic crust and abyssal lizardite-serpentinites in the slab. These three lithologies have distinct Br/Cl and I/Cl ratios (Fig. 3a). Typically, incoming plate sediments have very high Br/Cl and I/Cl ratios (up to 0.1), whereas abyssal lizardite-serpentinites have Br/Cl ratios similar to those of seawater and the primitive mantle (~0.003) and I/Cl ratios between those of the primitive mantle (0.0002) and pelagic sediments (0.001). The Br/Cl and I/Cl ratios of altered MORB cover relatively large ranges (0.0007 to 0.004 and 0.000003 to 0.0004, respectively)^39^, extending to values much lower than those of incoming sediments and abyssal serpentinites (Fig. 3a). The Br/Cl and I/Cl ratios of the forearc DR serpentinites overlap with those of altered MORB, and they are lower than those of incoming plate sediments (Fig. 3a), suggesting a more important contribution from dehydrating oceanic crust than from sediments. Halogen loss from the dehydrating crust is consistent with previous reports of lower Br and Cl concentrations in blueschist-facies metabasites from Turkey relative to pre-subducted altered oceanic crust^40^. Our interpretation is also consistent with the tectonic setting of the Dominican Republic in which the majority of sediments overlying the young oceanic lithosphere are accreted instead of subducted. Indeed, geophysical modeling confirms minimal metasediments in the subduction channel of the Dominican Republic^41^. On the contrary, lizardite-serpentinites from other forearc mantle wedges, such as Mariana and Guatemala, have Br/Cl and I/Cl ratios that are elevated with respect to abyssal lizardite-serpentinites (Fig. 3b), reflecting significant contribution from incoming plate sediments. This suggests dehydrating sediments make a considerable contribution to the composition of slab fluids in these particular subduction zones. Indeed, Mariana and Guatemala witness the subduction of old oceanic plates in which all incoming sediments are subducted instead of accreted^42^.

Fluorine contents of the DR forearc mantle lizardite-serpentinites are slightly elevated relative to the PM and DMM and are similar to those of Mariana forearc mantle serpentinites^6^ (Fig. 2c). We consider the most likely source of F in these forearc mantle serpentinites to be F expelled from sediment pore fluids during their early (<15 km) subduction. Structurally-bound F, on the other hand, likely remains in sediments during early subduction due to the high thermal stability of F-bearing amphibole and mica^43^ and the ability of F^−^ to substitute for O^2−^ in nominally anhydrous minerals^32^. It also seems unlikely that F is sourced from abyssal serpentinites since the average F content of the subducted serpentinites is actually higher than that of the obducted samples. The retention of structurally-bound F during subduction is supported by low (0.02–0.42) partitioning coefficients of F between water and hydrous minerals^44^ and mass balance calculations that predict ~95% of subducted F is not recycled to volcanic arcs^1^.

Elevated B concentrations in the DR forearc lizardite-serpentinites are likely sourced from dehydrating sedimentary and mafic rocks of the subducting slab during progressive metamorphism^45,46^. Perhaps, some B may be derived from subducting serpentinites during lizardite-antigorite transition^5,9^. However, the high B concentrations in the DR antigorite-serpentinites and in HP serpentinites of the Ligurian Alps, Italy^11^, suggest the retention of significant B in the slab during its subduction.

### Deep volatile cycling

Slabs are much cooler than the mantle wedge at a given depth, and as such, serpentinites in the slab are stable to greater depths than serpentinites in the mantle wedge. Thermal models have shown that abyssal antigorite-serpentinites dehydrate at depths of 80 to 150 km in warm subduction zones^47^ but may be stable to depths greater than 200 km in cool subduction zones^34^. Therefore, they may have the capacity to transport fluids and FME beyond slab depths associated with most volcanic arcs.

The high F and B contents and F/Cl and B/Cl ratios of the antigorite-serpentinites suggest F and B are, at least partially, retained in the slab during subduction. These findings support previous estimates of low fractions of these elements returning to the surface through volcanic front magmas. For example, Straub & Layne (2003a)^1^ calculate ~4–5% F and ~77–100% Cl entering subduction zones are discharged through volcanic front magmas at the Izu arc, and Moran *et al*. (1992)^48^ suggest a maximum of 62% of subducted B is returned to the surface via arc magmatism. However, neither of these calculations consider the significant input of volatiles from subducting abyssal serpentinites, nor do they account for volatile reflux back to the surface along the subduction interface. Therefore, these estimates may overstate the true fraction of FME incorporated into arc magmatism from the subducting slab.

In addition to antigorite, significant F and B may also be retained in the slab in minerals such as apatite (F) and phengite (F, B) that are stable to depths of 200 and 300 km, respectively^34,49^. Indeed high F contents have been reported in apatite (up to 3.5 wt%) and phengite (up to 570 ppm) of lawsonite blueschists^40^, and high B (~100 ppm) has been documented in phengite of blueschist facies metasedimentary rocks^50^. Fluids and FME may also be retained to depths of 300 km in hydrous phase-A after antigorite breakdown^34^. Even after the complete decomposition of the aforementioned minerals, a significant portion of FME may be retained in the slab by nominally anhydrous phases. Experimental studies have shown the accommodation of F and B in olivine, pyroxenes and garnet under high pressures and temperatures^32,51–53^, and high F (up to 130 ppm) and B (up to 20 ppm) have been reported in secondary olivine after serpentine^54^. The retention of F in slabs may contribute to its high concentration in deep-seated magmas, such as ocean island basalts^22^.

Compositions of the DR antigorite-serpentinites, and compositions of metabasites and metasedimentary rocks in slabs from elsewhere, suggest F and B are likely retained in the slab to great depths, while others, such as Cl, Br and I, have much shallower cycling in subduction zones.

## Methods

Halogens are extracted from 0.5 g of finely ground sample material using a pyrohydrolysis technique with the accuracy of the technique verified by the analysis of four reference standards (BCR-2, JB-1, JB-3 and MRG-1)^40^. Analyses of F and Cl are carried out using a Dionex ICS-2100 ion chromatography system equipped with KOH eluent generator at the University of Ottawa. Instrumental detection limits (3σ) for F and Cl are 0.053 ppm and 0.042 ppm, respectively. Analyses of Br (79Br) and I (127I) are carried out using an Agilent Technologies 7700 series inductively coupled plasma-mass spectrometer (ICP-MS) at the University of Ottawa. Samples are diluted 5x to minimize the effects of Na precipitate, and 1% NH_3_ is added to stabilize the anions in solution. Instrumental detection limits (3σ) for Br and I are 0.028 and 0.14 ppb, respectively. The reagent grade V_2_O_5_ (Elemental Microanalysis Ltd.) used as a catalyst for the extractions contains 0.36 ppm F, 0.004 ppm Cl, 32 ppb Br and 2.7 ppb I after drying at 325 °C for 24 h. The method blank, as determined by repeat extractions without sample or catalyst, is 0.011 ppm F, 0.108 ppm Cl, 4.1 ppb Br and 2.7 ppb I. Average standard deviations (1σ) of repeat halogen extractions (n = 2 or 3) are 8.93% for F, 14.9% for Cl, 30.6% for Br and 12.4% for I. Bulk B concentrations were determined on 1.0 g of finely ground sample material by Activation Laboratories (Ancaster, Ontario, Canada) by prompt gamma neutron activation analysis (PGNAA) with a 2 ppm detection limit. Accuracy of the technique was verified by analysis of two geologic reference materials (SY-2 and SY-3).

### Data availability

The datasets generated and analyzed during the current study are available from the corresponding author within a reasonable time-frame, upon request.

## Electronic supplementary material

Supplementary Information
