# Supplementary material for: Tracing halogen and B cycling in subduction zones based on obducted, subducted and forearc serpentinites of the Dominican Republic
Source: Sci Rep. 2017 Dec 19;7:17776. doi: 10.1038/s41598-017-18139-7 (PMC5736666; doi:10.1038/s41598-017-18139-7)
Supplement: Supplementary file 1 — Supplementary Information [file 41598_2017_18139_MOESM1_ESM.pdf]

## Supplement to:

# Tracing halogen and B cycling in subduction zones based on obducted, subducted and forearc serpentinites of the Dominican Republic

Lilianne Pagé[1]* & Keiko Hattori[1]

[1]Department of Earth and Environmental Sciences, University of Ottawa, Ottawa, Canada, K1N 6N5 (*correspondence: lpage097@uottawa.ca)

Table S1. Summary of the occurrences and mineralogy of serpentinites from the northern Dominican Republic as modified from Saumur et al. (2010).

| Geologic context | Location | Samples[a] | Serpentine phase | Accessory minerals[b] | Protolith |
|---|---|---|---|---|---|
| 'Northern Terrane' ophiolite complexes | Puerto Plata complex | 4, 6a, 31 | Lizardite | Mag, Chr | Obducted abyssal |
| | Gasper Hernandez | 8b | Lizardite | Mag, Chr | Obducted abyssal |
| Mélanges | Arroyo Sabana | 87, 89 | Antigorite | | Subducted abyssal |
| | Jagua Clara | 21a, 91, 6-50c, 6-52a, 6-54a, 94* | Antigorite (+ lizardite*) | 1°Ol, 1°Opx | Subducted abyssal |
| Fault zones | Camú | 9a, 18b, 6-80, 6-81 | Lizardite | Mag, Chr | Forearc mantle |
| | Septentrional | 34a, 34c, 68, 6-36a, 6-36c<br>Rio Cuevas: 45, 48, 60 | Lizardite | Mag, Chr<br>1°Ol,<br>1°Opx | Forearc mantle |

[a] Prefix of RD is omitted.
[b] Abbreviations: Mag – magnetite, Chr – chromite, 1°Ol – primary olivine, 1°Opx – primary orthopyroxene

*Sample petrography*

Serpentine species were previously identified using micro-Raman spectrometry and X-ray powder diffraction (ref. 1). Samples of the Northern Terrane and fault zones are composed of hourglass-textured lizardite pseudomorphs after olivine, along with minor, dusty magnetite. Samples of the tectonic mélanges are comprised of blades of antigorite overprinting the original rock/mineral textures, along with fine-grained magnetite. Mélange sample RD94 consists of pseudomorphic lizardite replacing olivine and minor, penetrative antigorite blades. Primary olivine is preserved in fault-zone sample RD68, and mélange sample RD94, while primary orthopyroxene is identified in fault-zone samples RD18b, RD68, RD34 and mélange sample RD94.

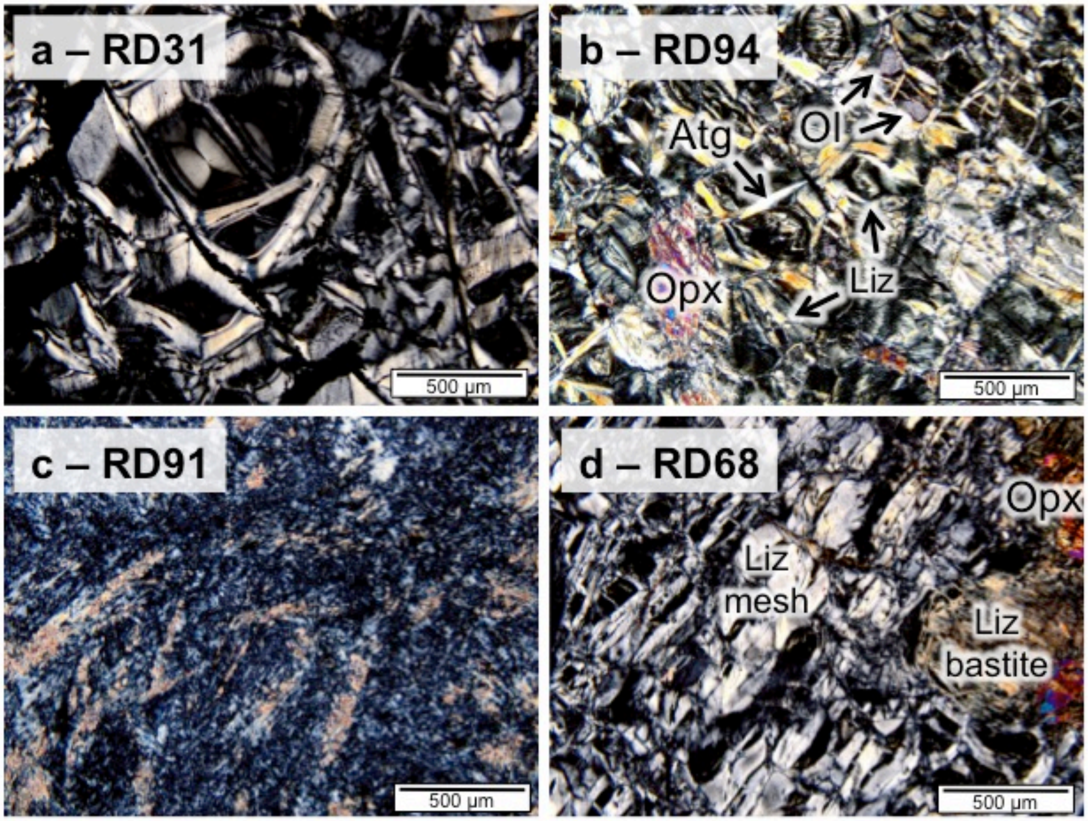


Figure S1. Photomicrographs of serpentinite samples under crossed polars. (a) Pseudomorphic lizardite with hourglass texture in obducted abyssal sample RD31. (b) Antigorite blades cross-cutting pseudomorphic lizardite and relic primary olivine and orthopyroxene in subducted abyssal sample RD94. (c) Antigorite blades in antigorite-rich matrix in subducted abyssal sample RD91. (d) Lizardite mesh after olivine and lizardite bastite after orthopyroxene in forearc mantle sample RD68. Atg – antigorite, Liz – lizardite, Ol – olivine, Opx – orthopyroxene

*Protolith and location of samples in subduction zone*

The Dominican Republic serpentinite samples are classified into three types: i) obducted abyssal ii) subducted abyssal, and iii) forearc mantle serpentinites. Bulk rock and Cr-spinel compositions were used to determine the protolith (abyssal versus forearc mantle peridotite) of each sample (ref. 1). Serpentinites along the fault zones have more refractory protoliths than those of the Northern Terrane and tectonic mélanges, as indicated by higher MgO (up to 40 wt%) and lower $Al_2O_3$ (< 0.7 wt%) content. The fault-zone serpentinites plot within the field of forearc mantle peridotites on the diagram of Al/Si versus Mg/Si, whereas the samples from the Northern Terrane and mélanges plot within the abyssal peridotite field (ref. 1). The platinum group element (PGE) contents of the fault-zone samples also support a highly refractory protolith, with an enrichment of Iridium-group PGEs (Ir, Os and Ru) and a depletion of Palladium-group PGEs (Pt, Pd and Rh). Furthermore, the high Cr# (up to 0.7) of spinel grains in the fault-zone

serpentinites suggest a forearc mantle origin, since abyssal peridotites formed a slow spreading ridges typically contain spinel with a much lower Cr# (<0.4; ref. 2).

The tectonic mélanges represent a serpentinite-rich subduction channel, as suggested by the occurrence of eclogitic fragments in the mélanges (ref. 3), and high-temperature antigorite overprinting pre-existing lizardite in the serpentinite samples. In contrast, the serpentinites of the Northern Terrane ophiolite complex are comprised of low-temperature lizardite, supporting that they have not undergone subduction.

## References for Supplement


1. Saumur, B. M., Hattori, K. H., & Guillot, S. Contrasting origins of serpentinites in a subduction complex, northern Dominican Republic. *Geol. Soc. Am. Bull.* **122(1-2),** 292-304 (2010).
2. Michael, P. J., & Bonatti, E. Peridotite composition from the North Atlantic: regional and tectonic variations and implications for partial melting. *Earth Planet. Sci. Lett.*, **73(1),** 91-104 (1985).
3. Krebs, M. *et al*. The dynamics of intra-oceanic subduction zones: a direct comparison between fossil petrological evidence (Rio San Juan Complex, Dominican Republic) and numerical simulation. *Lithos*, **103(1),** 106-137 (2008).
